# The Recrudescence of Quarantine

**Published:** 1898-01-08

**Authors:** 


### The Hospital

A Journal of

The Medical Sciences and Hospital Administration.

Vol. XXIII.—No. 58[illegible].] Edited by Sir Henry Burdett, K.C.B., and Solomon C. Smith, M.D., M.R.C.P. [Jan. 8, 18[illegible]8.

### The Recrudescence of Quarantine.

It is not a little curious, at this somewhat advanced period of the history of civilisation, to find that proposals for the more or less complete restoration of quarantine, as a protection against the introduction of disease, are not only received with favour by non-medical legislative bodies and by certain sections of the general public, but are advocated, and to some extent enforced, by direct medical authority. We have heard of so-called "quarantine camps" as part of the organisation called into existence for the purpose of checking the advance of bubonic plague in Bombay; and the *New York Medical News*, of so late a date as Christmas Day, contains not only the full text of Senator Caffery's Bill for the more strict enforcement of quarantine in the United States with reference to yellow fever, but also, what is infinitely more curious in a medical journal, a leading article commendatory of the course proposed, in which it is said that Congress has before it for enactment this winter no measure of more pressing urgency than this Quarantine Bill introduced by Senator Caffery. It does not surprise us that an ignorant population should stand on guard at railway stations with loaded fire-arms, and should forbid trains to stop or passengers to alight, but it does surprise us to find a medical contemporary even appearing to admit that, "the paper plausibilities of quarantine" are able to confer some kind of degree of additional security upon States in the vicinity of those which may be visited by a yellow fever epidemic.

The belief that any such security can be afforded in the manner indicated is one which could hardly fail to spring up and flourish during the darkness of the Middle Ages. The first proposals for quarantine date from the middle of the fourteenth century, and originated in the city of Milan, as a precaution against the Black Death. The example thus set was followed in Venice, where the first *lazaretto* was established in 1423, the disease then to be kept at bay being bubonic plague. Two centuries later the system was almost universal and had reached its full development, insomuch that very elaborate regulations were formed and enforced in this country with reference to the plague which appeared so early as in 1636, and which committed such terrible ravages in London and in some country districts, as at Eyam, between 1663 and 1666. These endeavours to exclude plague were as effectual, in the words of Sir John Simon, "as if their intention had been to bar out the east wind or the new moon;" but, notwithstanding this, the epidemic of cholera which prevailed in Europe in 1831 found not only the populace, but even the sanitary authorities of this country, prepared to trust in quarantine as their supreme hope. As the Government could only control the regular channels of trade or passage, all persons of influence resident on the coast, and particularly in retired villages, were urged to impress upon their neighbours the dangers of intercourse with smugglers and other evaders of quarantine. It might have been thought that this very injunction would of itself have been sufficient to prove to those who issued it the utter futility of the whole proceeding. The Government was able to interfere just so much as to cause the maximum of inconvenience and loss to healthy people, and the maximum of injury to trade; and, when this was done, they were unable to touch so much as the fringe of the innumerable points of leakage, which even the best organised system of quarantine must leave wholly unprovided for. Notwithstanding the quarantine, the disease was not only introduced, but it spread with terrible rapidity, and produced a mortality of many thousands, the precise amount of which it would now be impossible to ascertain. Taught by experience, the General Board of Health, in 1849 and in 1852, strenuously pointed out that quarantine could not give any but a false security for the purpose it pretended to accomplish; and, adducing illustrations of its futility and oppressiveness as commonly administered, boldly proposed, as a practical conclusion, that this country should entirely set aside its existing quarantine establishments, and should rely exclusively upon the protection it could derive from a system of local sanitary improvements. Our present method is to admit disease freely, but to be on the watch for it when it comes. If plague or yellow fever were brought to any English port, the actual sick would be landed and placed in a proper hospital for the reception of infectious cases; the sound would be permitted to proceed to their several destinations, the sanitary authorities of which would be instructed to keep them under observation until all danger was past, and to send them to hospital if the disease should show itself in them; and the ship and its cargo would be subjected to disinfection. When we had quarantine, plague and cholera were not only introduced, but destroyed their thousands. During the last European epidemic, cholera was introduced into many of our ports, and it fizzled out as harmlessly as a lighted match on a stone floor.

